# Assessment and Management of Atrial Fibrillation in Older Adults with Frailty

**DOI:** 10.3390/geriatrics9020050

**Published:** 2024-04-15

**Authors:** Andrea Nathalie Rosas Diaz, Aaron L. Troy, Vladimir Kaplinskiy, Abiah Pritchard, Rati Vani, Darae Ko, Ariela R. Orkaby

**Affiliations:** 1 Beth Israel Deaconess Medical Center, Boston, MA 02215, USAatroy1@bidmc.harvard.edu (A.L.T.);; 2 Section of Cardiovascular Medicine, Boston Medical Center, Boston University Chobanian and Avedisian School of Medicine, Boston, MA 02118, USA; 3 Hinda and Arthur Marcus Institute for Aging Research, Hebrew SeniorLife, Harvard Medical School, 1200 Center Street, Boston, MA 02131, USA; 4 New England GRECC (Geriatric Research, Education and Clinical Center), VA Boston Healthcare System, Boston, MA 02130, USA; 5 Division of Aging, Brigham & Women’s Hospital, Harvard Medical School, Boston, MA 02115, USA

**Keywords:** frailty, atrial fibrillation, cardiovascular disease

## Abstract

Atrial fibrillation (AF) is a major driver of morbidity and mortality among older adults with frailty. Moreover, frailty is highly prevalent in older adults with AF. Understanding and addressing the needs of frail older adults with AF is imperative to guide clinicians caring for older adults. In this review, we summarize current evidence to support the assessment and management of older adults with AF and frailty, incorporating numerous recent landmark trials and studies in the context of the 2023 US AF guideline.

## 1. Introduction

Atrial fibrillation (AF) is a major driver of morbidity and mortality among older adults with frailty [1,2,3,4]. AF increases the risk of stroke, heart failure, cognitive impairment and dementia, chronic kidney disease, and mortality [1]. The array of adverse outcomes underscores the need for a timely diagnosis and effective management in older adults with frailty.

The prevalence of AF is projected to increase to 12.1 million in 2030 in the United States and 14 million by 2060 in Europe [1,2,3,4,5]. As the global population ages, rising rates of both cardiovascular disease and geriatric conditions are increasingly recognized as major public health issues [4]. Frailty, a clinical syndrome characterized by increased vulnerability to stressors due to decreased physiological reserve, is prevalent among older adults with AF [3]. However, given the low frequency of frailty assessment in AF trials, many guideline recommendations for AF do not sufficiently account for the clinical complexities of older adults with frailty, which impacts changes in pharmacokinetics and pharmacodynamics, life expectancy, and patient preferences. Indeed, ACC/AHA/ACCP/HRS recently published a paradigm-shifting Guideline for the Diagnosis and Management of Atrial Fibrillation (2023 US AF guideline) that emphasizes the importance of shared decision-making but does not directly address frailty due to the paucity of practice-guiding data [1,2].

Understanding and addressing the needs of frail older adults with AF is thus imperative to guide clinicians caring for older adults. In this review, we summarize current evidence to support the assessment and management of older adults with AF and frailty, incorporating numerous recent landmark trials and studies in the context of the 2023 US AF guideline.

## 2. Frailty and Atrial Fibrillation Risk

The estimated prevalence of AF in older adults with frailty varies from 48–75% [6]. Conversely, the estimated prevalence of frailty in older adults with AF ranges from 6 to 71% [7,8]. The wide variation is driven in part by differing sample populations and definitions of frailty across studies.

**Figure 1:**
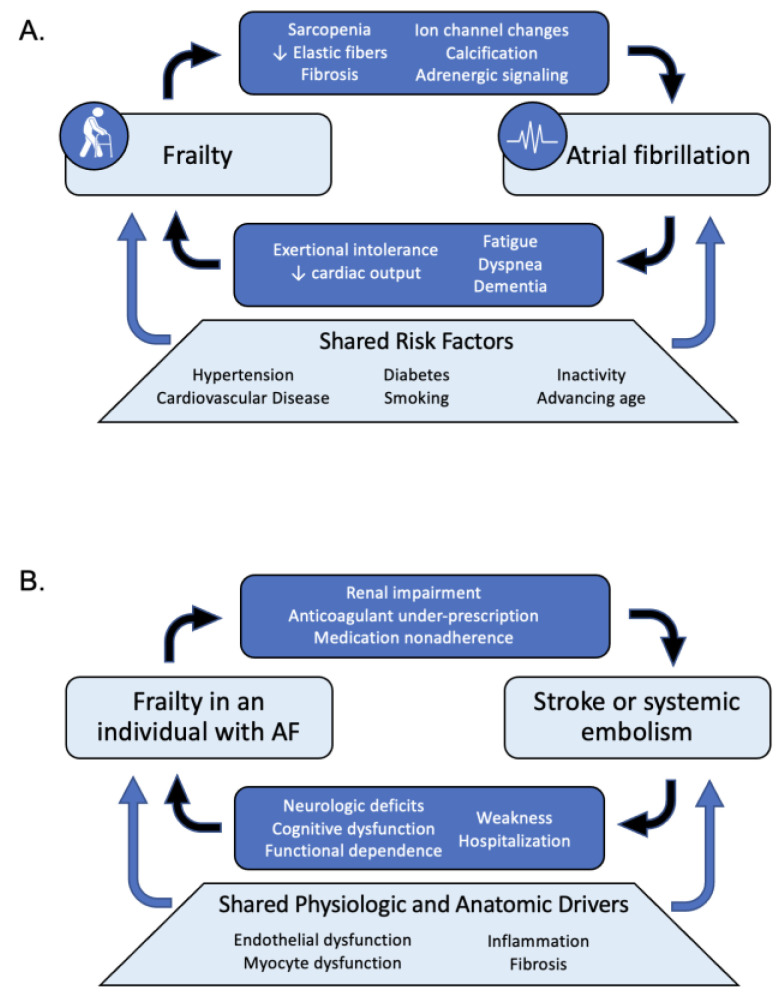
The interplay between risks of frailty, AF, and stroke. (**A**) depicts the shared risk factors and meditators of the bidirectional relationship between frailty and atrial fibrillation, with arrows indicating direction of effect. (**B**) similarly depicts the shared risk factors and mediators of the bidirectional relationship between frailty and stroke or systemic embolism in an individual with clinical AF.

The high prevalence of AF in older adults with frailty is related to both shared risk factors as well as a hypothesized bidirectional relationship whereby frailty increases the risk of AF and vice versa (Figure 1) [9]. Several studies have shown that well-established cardiovascular disease risk factors, including hypertension, diabetes, smoking, physical inactivity, and poor nutrition, increase the risk of both AF and incident frailty [10,11,12].

Although the mechanisms underlying AF remain active areas of study, biologic aging, captured by frailty, drives many anatomic and physiologic changes in the heart that are hypothesized to increase AF risk (Figure 1A). On a physiologic level, AF is driven in part by fibrosis and other structural changes to the atria that promote the development of numerous reentrant ectopic foci in both the atria and the pulmonary veins [13,14]. Aging is associated with myocardial remodeling—including decreased cardiomyocyte and elastic fiber volume, increased myocardial fibrosis, increased arterial stiffness, and diastolic left ventricular dysfunction—all of which may predispose the atria to conduction abnormalities and fibrillation [15]. Pre-clinical studies have also demonstrated delayed interatrial conduction and longer p-wave duration associated with age [16]. Even changes in the autonomic nervous system, from increased norepinephrine levels to decreased beta-adrenergic receptor signaling changes in adrenergic receptors and downstream signaling, have been shown to accumulate with age and may promote AF [17].

Conversely, AF also increases the risk of frailty, with data suggesting AF is associated with frailty independent of age, sex, and comorbidities, including cardiovascular disease risk factors [18]. AF causes dyspnea, fatigue, and other symptoms associated with impaired cardiac output, which limit physiologic reserve and promote a sedentary lifestyle, thereby driving weakness and sarcopenia [18,19]. AF is also associated with an increased risk of dementia, even after adjustment for comorbidities and stroke [20]. Additionally, medications used to treat AF can lead to frailty-defining symptoms, such as weakness and lethargy from direct effects of rate and rhythm control agents, complications of anticoagulants, and polypharmacy.

Finally, AF-related thromboembolic stroke and frailty have an independent bidirectional relationship that warrants its own discussion (Figure 1B). Physiologically, AF and the resultant uncoordinated contraction of the atrial myocardium allows for stasis of blood, primarily in the left atrial appendage, which promotes the formation of clots that can embolize and cause stroke. More recently, physiologic abnormalities, including endothelial pathology, fibrosis, and myocyte dysfunction, have been identified as additional mediators of this increased risk of stroke [21,22]. Frailty increases the risk of stroke in AF [7,23], driven in part by physiologic changes such as inflammatory cytokines and renal impairment [24] and lower utilization of anticoagulants [7,23,25]. Moreover, older adults with frailty who have strokes have longer post-stroke hospitalization and a higher risk of death than those without frailty [26,27,28].

## 3. Frailty Assessment for Older Adults with Atrial Fibrillation

Frailty is a multisystem syndrome of decreased physiological reserve against stressors that can be assessed using multiple validated approaches [29,30]. There are two leading definitions: the physical phenotype and the cumulative deficit model. The physical phenotype developed by Fried and colleagues is comprised of five items: slow walking speed, unintentional weight loss, self-reported exhaustion, low physical activity, and low grip strength [30]. Rockwood and colleagues developed the cumulative deficit model, which counts deficits in health across multiple domains, including physical function, cognition, morbidities, nutritional deficits, mental health, and geriatric syndromes, based on the Comprehensive Geriatric Assessment [31]. These two leading theories of frailty, one focused on physical function and the other a more holistic count of vulnerabilities in health an older adult may experience, have led to over 60 tools that can be used clinically to measure frailty [32]. Briefer tools, such as 4-m gait speed or the Clinical Frailty Scale, a 9-point scale that assesses functional and health status in the 2 weeks prior, can be readily incorporated into routine clinical practice [32,33].

Multiple AF studies have examined the role of frailty [34]. A prospective study in China compared the physical phenotype and cumulative deficit approach in adults ≥65 years old admitted to the hospital and with a diagnosis of AF [35]. Frailty ranged from 34.5% using the physical phenotype to 42.6% using the cumulative deficit model. Malnutrition and polypharmacy were independent predictors of frailty in this older AF cohort. Wilkinson and others estimated the prevalence of frailty in participants of the ENGAGE AF-TIMI 48 trial. One in five subjects in ENGAGE AF-TIMI 48 were found to be frail using a cumulative deficit approach, with 17.8% of subjects having mild-moderate frailty and 1.7% having severe frailty [36]. Investigators prospectively assessed phenotypic frailty in the ELDERCARE-AF trial and classified 40.9% of study participants as frail [37]. In a systematic review and meta-analysis, the prevalence of frailty in adults with atrial fibrillation ranged from 30% to 50%. Subgroup analysis showed that the prevalence of frailty was higher in studies that used the clinical frailty scale [38]. Frailty was also assessed using a cumulative deficit approach in a prospective multicenter European observational registry, in which 21% of study participants were identified to be frail [39].

Other indices have been used to measure frailty in adults with AF. A prospective study in Australia used the Edmonton Frail Scale to identify frail participants to assess differences between frail and non-frail adults ≥ 65 years with AF admitted to the hospital. Frail older adults with AF were older, had more comorbidities, were more likely to be nursing-home-dwelling, had a higher prevalence of HF, peripheral vascular disease, malnutrition, and depression, and had longer lengths of stay [40]. The FRAIL-AF trial evaluated the safety of switching from a vitamin K antagonist to a DOAC in frail older adults with AF and used the Groningen frailty indicator score > 3 to determine eligibility [41]. Multiple retrospective studies have used claims-based frailty indices to assess frailty in patients with AF based on the deficit accumulation approach [42]. One assessed the effectiveness and safety of OAC among frailty older adults with AF in Korea, identified using the Hospital Frailty Risk Score. Individuals with frailty were older, more likely to be female, had higher CHA2DS2-VASc scores, and increased multimorbidity [25].

Given the prevalence of frailty in older adults with AF and the heightened morbidity, mortality, and management complexity in this population, we suggest that frailty assessment be incorporated into the evaluation of all older adults with AF in both clinical and research settings using any available validated frailty tool. A brief assessment of walking speed or the Clinical Frailty Scale can be readily implemented into clinical care for older adults with AF. Healthcare systems may consider employing claims-based frailty indices based on the cumulative deficit approach derived from electronic health record data. Clinical trials for AF therapies may consider incorporating the physical phenotype for prospective frailty assessment. Regardless of the approach, standardizing and increasing frailty assessment in older adults with AF is needed.

## 4. Atrial Fibrillation Assessment for Older Adults with Frailty

Given the increased risk and prevalence of AF in older adults with frailty, these individuals should be closely monitored for incident AF [1,2]. The 2023 US AF guideline re-classified AF into stages: Stage 1 signifying At-Risk for AF, Stage 2 Pre-AF, Stage 3A Paroxysmal AF, Stage 3B Persistent AF, Stage 3C Long-standing Persistent AF, Stage 3D successful AF ablation, and Stage 4 Permanent AF (Table 1) [1]. This new classification recognizes that individuals at each stage require different evaluation approaches; however, the impact of frailty at each stage is not addressed.

Screening strategies for AF include opportunistic screening, such as pulse palpation with reflex to ECG if irregular, or systematic screening with a more reliable modality. There are also multiple tools available to screen for AF, such as single-lead ECG, 12-lead ECG, continuous cardiac monitoring, and smartwatches. The most recent ESC guideline recommends opportunistic screening with pulse palpation in adults aged 65 years and older, but the U.S. Preventive Services Task Force (USPSTF) considers evidence to be insufficient to recommend any AF screening strategy in asymptomatic adults aged ≥ 50 [2,43].

Studies have demonstrated that multiple screening approaches increase the diagnosis of asymptomatic AF [44,45,46,47]. In the SAFE study, both systemic and opportunistic screening strategies increased new AF diagnosis rates, and both strategies were found to be cost-effective in adults aged ≥ 65 [44]. Pacemakers and defibrillators can detect AF and have been validated against ECG as a gold standard [1]. Positive signals from other modalities, such as pulse palpation or smartwatch single-lead ECG, should be confirmed with a 12-lead ECG or a single-lead ECG recording for ≥30 s, as studies have shown that approximately one of every three individuals with irregular rhythms detected on smartwatch will have confirmed AF on ECG [2,48]. Continuous ECG monitoring was tested in the LOOP Study, a randomized controlled trial of over 6000 individuals aged 70–90 with at least one stroke risk factor, in which AF screening using an implantable loop recorder was compared with usual care. Loop recorder screening was associated with a threefold increase in atrial fibrillation diagnosis but no reduction in the risk of stroke or systemic embolism [49].

**Figure 2:**
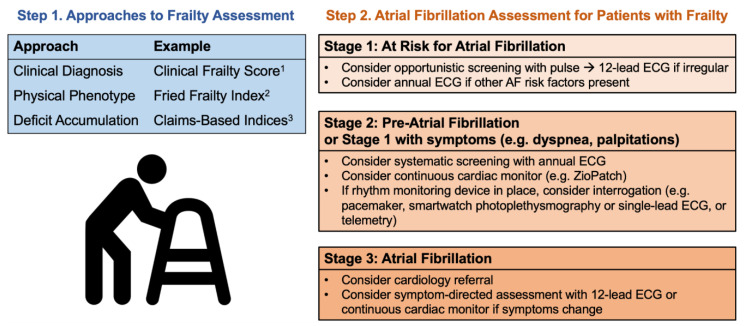
Frailty and Atrial Fibrillation Assessment in Older Adults. 1. Developed by Rockwood [31]. 2. Developed by Fried, et al. [31]. 3. Developed by Kim, et al. [50].

Given the increased risk of AF in older adults with frailty, the pre-test probability of detecting occult AF is higher than in non-frail older adults. Therefore, the benefit and cost-effectiveness of screening may be augmented in this population. As a result, we suggest that any older adult with frailty may be considered Stage 1 or at-risk, and opportunistic screening may be considered (Figure 2). For those with Stage 1 and additional AF risk factors, systematic screening with annual ECG may be reasonable. For older adults with Stage 2 pre-AF—evidence of structural or electrical findings predisposing them to AF—or Stage 1 with symptoms such as palpitations or dyspnea, clinicians may consider obtaining an annual ECG and, if negative, a one-time continuous cardiac monitor. If these patients have additional data available, such as from a smartwatch or telemetry, these can be examined during routine clinical evaluation. For patients at Stage 3, with established AF, cardiology referral may be considered, and those with new or evolving symptoms may benefit from 12-lead ECG or a continuous cardiac monitor to assess AF burden. Finally, in all older adults with an implantable cardiac device with an atrial lead, regular device interrogation is recommended regardless of AF stage [1,2].

During the screening process, clinicians can consider following an integrated care approach using the Atrial fibrillation Better Care (ABC) pathway [51,52]. For frail adults at risk of AF or considered to be pre-AF, efforts must be focused on risk factor management and comorbidities optimization. For patients diagnosed with AF, anticoagulation and symptom management (whether with rate or rhythm control strategies) should be prioritized. A European long-term registry studied if adherence to the ABC pathway decreases the risk of adverse outcomes in clinically complex patients. Patients were considered as being clinically complex if they had frailty, multimorbidity, and/or polypharmacy. Adherence to the ABC pathway decreased the risk of all cause death and MACE in clinically complex and frail patients [53]. Two ongoing projects, based in Europe, the AFFIRMO and EHRA-PATHS, aim to develop new pathways and an integrated approach in the assessment and management of adults with AF and multimorbidity [54,55].

In summary, opportunistic or systematic AF screening in older adults can increase the detection of AF, although data are needed to determine the cost-effectiveness of this approach. Evidence to guide screening strategies specifically in older adults with frailty is limited, and no studies have investigated outcomes associated with screening in this population. Future work studying the impact of AF screening on cardiovascular outcomes, such as rates of ischemic stroke, systemic embolism, and death, and on patient-reported psychological and financial outcomes are needed to guide practice and are underway [56]. However, given the increased risk of both AF and AF-related morbidity and mortality for older adults with frailty, increased screening can be considered for these individuals at earlier stages of AF.

## 5. Rate Control and Frailty

AF is often complicated by rapid ventricular rates, which can be associated with significant impairment and management challenges for older adults with frailty. Symptoms, such as lightheadedness, exertional intolerance and presyncope, and hemodynamic compromise, are particularly impairing for individuals with frailty, given their limited cardiopulmonary reserve. Moreover, adverse effects of rate control agents can be poorly tolerated in individuals with frailty, and alternative options, such as rhythm control, may be limited by multimorbidity and polypharmacy.

Trials comparing the efficacy of rate and rhythm control may not be generalizable to older adults with frailty, and subgroup analyses may yield limited and, at times, conflicting results. A multicenter randomized controlled trial of 205 individuals in Poland with AF showed parity between rate and rhythm control [57], recruited subjects with a mean age of 61 years, and required all participants to undergo several exercise tolerance tests. The landmark AFFIRM trial [58], published 22 years ago, favored rate control in 4060 patients with age ≥ 65 and known AF, with a non-significant increase in mortality over 3.5 years of follow-up. This differed from the overall primary endpoint analysis that demonstrated non-inferiority. Propensity-matched analysis in patients aged 65–80 [59] from the AFFIRM trial yielded similar results favoring rate control, with significantly lower rates of mortality and hospitalizations. It is notable that the rhythm control strategy did not include catheter ablation as a possible treatment option.

Older adults with frailty have also been underrepresented in the few trials assessing optimal heart rate goals and antiarrhythmic efficacy for AF rate control. RACE II [60], the sole randomized trial evaluating optimal ventricular rate target in AF, evaluated 614 patients with permanent (Stage 4) AF and limited its inclusion to adults up to 80 years. RACE II showed no difference in the primary composite outcome between lenient (<110 beats-per-minute) and strict (<80 beats-per-minute) rate goals. On the other hand, an observational study using the ORBIT-AF registry showed that a resting heart rate > 65 bpm and <65 bpm was associated with increased all-cause mortality, cardiovascular death, and adverse cardiovascular events in older adults with AF [61].

Rate control can be achieved with three major classes of medications: non-dihydropyridine calcium channel blockers (i.e., Verapamil, Diltiazem), beta-blockers (i.e., Metoprolol, Atenolol, Timolol, Pindolol, Nadolol, Propranolol, Bisoprolol, Carvedilol and, in the acute setting, Esmolol) and Digoxin (Table 2). Data on comparative efficacy is limited. The RATAF investigator-blinded crossover study [62] compared diltiazem, verapamil, metoprolol, and carvedilol in 60 patients with permanent AF with a mean age of 72 years and demonstrated increased efficacy of calcium channel blockers in both rate control and symptom reduction. Per the 2023 US AF guideline, however, beta blockers and calcium channel blockers are both adequate first-line therapies in older adults, barring other contraindications [1,63].

Beta-blockers and calcium channel blockers can be complicated by hypotension, which can lead to falls and other complications in individuals with frailty and is in part mediated by baseline decreased adrenergic receptor signaling with advanced biologic aging. Orthostatic hypotension is extremely common in older adults, with prevalence of up to 68% among geriatric inpatients [63]. In those with heart failure, studies have demonstrated that frailty is associated with a lower rate of guideline-directed beta blocker prescription [64], related to concerns about adverse effects and perhaps actual risks of over-medication in this group [65].

Digoxin does not precipitate hypotension but has other limitations, including less effective rate control in individuals with high catecholamine states and a narrow therapeutic window [66]. Subgroup analyses of the DIG study, a randomized controlled trial that examined the efficacy of Digoxin in 631 patients with heart failure and no AF, yielded conflicting results in older populations, with one study demonstrating an increased risk in 30-day hospitalization in patients with heart failure with preserved ejection fraction [67] and another demonstrating a decrease in 30-day hospitalizations in older subjects with heart failure with reduced ejection fraction [68]. The Beers Criteria, a guideline of high-risk medications to use with caution or avoid in older adults, recommends avoiding Digoxin as the first line and, if used, avoid dosages > 0.125 mg/day [69]. In the absence of heart failure, some studies have also suggested an increased risk of mortality with digoxin use [70], though literature on this is conflicting, and digoxin remains a recommended pharmacotherapy in the 2023 US AF guidelines.

In older adults with frailty for whom attempts at ventricular rate control have been unsuccessful and sinus rhythm cannot be restored, AV node ablation with pacemaker implantation or cardiac resynchronization (CRT) remains an important therapeutic option. Several small randomized controlled trials have demonstrated that this approach yields improved quality of life [71,72]. Among patients with long-standing AF, no difference in all-cause mortality was found between CRT and right ventricular pacing [72].

This must be weighed against the risks of lifelong pacemaker dependence, which has higher complication rates in those ≥ 75 [73]. Advancements in pacemaker technology, including leadless devices, may broaden indications of this approach.

Overall, evidence supports lenient rate control (<110 beats-per-minute) for older adults with AF, starting with a low-dose calcium channel blocker or beta blocker to improve symptoms and hemodynamics. However, compared with rhythm control and anticoagulation, few studies of rate control are generalizable to older adults with frailty, who have a higher risk of complications from both rapid rates and antiarrhythmic therapy. Future studies of rate control agents, heart rate targets, and complications in individuals with AF and frailty are strongly warranted.

## 6. Rhythm Control and Frailty

Given the potential risks of bradycardia and hypotension and limited data to support rate control for individuals with frailty and AF, rhythm control represents an important early consideration for these patients. Although limited, there is a growing evidence base to guide the choice of a rhythm control strategy, selection of antiarrhythmic medication, and consideration of referral for AF ablation for older adults with frailty.

Studies of AF rhythm control in older adults with frailty have demonstrated reassuring safety profiles and varying degrees of efficacy. A systematic review and meta-analysis of rate and rhythm control outcomes in adults ≥ 65 demonstrated no significant difference in all-cause mortality between rate and rhythm control strategies [74]. In addition, the EAST-AFNET 4 randomized controlled trial of early rhythm control vs. usual care in older adults found that rhythm control decreased the composite of death from cardiovascular causes, stroke, hospitalization for worsening heart failure, and acute coronary syndrome [75].

The choice of antiarrhythmic medication can be challenging for frail older adults, given multimorbidity, polypharmacy, and altered pharmacokinetics that can increase the risk of adverse effects [52]. Common contraindications for antiarrhythmic drugs in older adults include structural heart disease, QT prolongation, and renal failure [76]. Amiodarone is the most commonly used antiarrhythmic for AF and is the most effective at maintaining sinus rhythm compared with sotalol, dronedarone, propafenone, and flecainide (Table 3) [1,52,76]. However, the Beers Criteria recommends avoiding amiodarone as first-line therapy in patients without heart failure or substantial left ventricular hypertrophy [69]. As an alternative to antiarrhythmic medications, providers could consider catheter ablation. The 2023 US AF guideline supports catheter ablation for individuals with symptomatic AF if antiarrhythmic medications are not tolerated or contraindicated [1]. Retrospective studies have demonstrated a reassuring safety profile for ablation in older adults in general, with low rates of procedural complications across catheter technologies; however, data are limited among those with frailty [77,78,79]. In older adults, procedural times were longer and had a higher rate of non-pulmonary vein trigger sites [77]. A catheter ablation strategy does not increase the risk of mortality and adverse events in this population [78].

Regarding efficacy, the CABANA trial compared catheter ablation with antiarrhythmic medications and found no difference in the primary composite endpoint of death, disabling stroke, serious bleeding, or cardiac arrest but demonstrated the benefit of ablation for the secondary endpoints of AF recurrence and composite of death and cardiovascular hospitalization [78]. However, subgroup analyses revealed that the benefits of ablation were primarily observed in patients aged < 65. Similarly, a retrospective cohort study of nearly 200,000 patients aged ≥ 75 with AF in Korea found no difference in death or the primary composite outcome of death, heart failure admission, thromboembolism, or cardiac arrest among patients with frailty but did find benefit among non-frail patients [80]. However, in the CASTLE AF trial, adults with AF and HF who underwent catheter ablation had a lower rate of death or hospitalization for worsening heart failure [81]. A more recent retrospective study of over 20,000 patients ≥ 65 with AF, mostly in the US, reported a reduced risk of mortality in patients who underwent catheter ablation [82]. 

Overall, the evidence for early rhythm control in patients for AF is growing, and individuals with frailty may experience similar benefits to those without frailty. Despite its well-known side effect profile, amiodarone remains an important antiarrhythmic medication to consider given its efficacy, lack of proarrhythmic effect, and lack of contraindication in patients with comorbid structural heart disease. Given the challenges antiarrhythmic medications pose in this population, catheter ablation should also be considered, as early evidence suggests a robust safety profile. However, the efficacy of ablation among older adults with frailty remains unclear. Further studies comparing contemporary rhythm control strategies in older adults with AF will be required to guide practice for this high-risk population, particularly those with frailty.

## 7. Stroke Prevention and Frailty

Selecting a stroke prevention strategy in older adults with AF and frailty is a challenging and high-stakes clinical decision, given the elevated risks of both thromboembolism and hemorrhage, particularly in those with frailty who are already at increased risk of stroke and bleeding independent of AF and anticoagulation selection [83,84,85,86]. There is a robust and growing evidence base to guide anticoagulant selection in older adults with frailty, while more nascent evidence is emerging for other novel stroke prevention approaches for patients with high bleeding risk.

Oral anticoagulation with a direct oral anticoagulant (DOAC), or warfarin if DOACs are contraindicated, is recommended for prevention of thromboembolism in individuals with ≥2% annual risk of thromboembolism, regardless of AF pattern or stage [1]. Validated risk scores are useful for assessing thromboembolic risk, although none are specific to those with frailty. Most scores include age and multimorbidity; for example, the CHA_2_DS_2_-VASc score assigns 1 point for age 65–74 and 2 for age ≥ 75, as well as 2 points for history of stroke or TIA, and 1 point each for congestive heart failure, hypertension, diabetes, or female sex [87]. Anticoagulation is therefore indicated for all individuals with AF and age 75 and above and should be considered for those 65 and above, as the 2023 US AF guideline recommends anticoagulation for individuals with at least two non-sex-related points and consideration of anticoagulation for those with one non-sex-related point [1]. The 2023 US AF guideline emphasizes that bleeding risk scores should be used to identify modifiable bleeding risk factors, not to exclude patients from oral anticoagulation.

Given the elevated risk of stroke and bleeding in older adults with frailty, shared decision-making with patients and families is essential, including the support of evidence-based decision aids when possible. Although individual patient factors should be weighed carefully in these discussions, clinicians should clearly communicate that multiple studies have demonstrated the stroke protective benefit of oral anticoagulants to outweigh their associated bleeding risks across frailty and fall-risk categories [7,25,83,88]. Specifically, anticoagulants lower mortality among older adults with AF and frailty, and perhaps more important to patients and their families, lower the risk of stroke, with the stroke-protective benefits outweighing the risk of hemorrhage on a population level [7,25,83]. Despite robust evidence of anticoagulants’ net benefit for individuals with AF and frailty, frail older adults are less likely to be prescribed oral anticoagulants across healthcare settings, with frailty and falls cited as the most common reasons for nonprescription [42,88,89]. However, many older adults have no evidence-based contraindications to anticoagulation and, therefore, carry unnecessarily increased stroke risk driven in part by clinicians’ fear of bleeding [90,91].

Recent evidence has emerged to guide anticoagulant selection for older adults with frailty. A large retrospective cohort study comparing DOACs and warfarin found that, in older adults with AF and frailty, DOACs are associated with a lower risk of mortality, stroke, and bleeding [25]. Multiple large retrospective studies have demonstrated that apixaban carries a lower risk of clinical events—specifically a composite of stroke, systemic embolism, major bleeding, or death—compared with rivaroxaban, dabigatran, and warfarin in older adults with frailty [92,93].

Although observational data favors the initiation of a DOAC over warfarin for older adults with AF and frailty, the recent FRAIL-AF trial suggests that those already on warfarin should continue rather than switch to a DOAC [41]. In the multicenter randomized controlled trial, 1330 older adults with frailty, nonvalvular AF, and GFR > 30 who were on INR-guided warfarin therapy were randomized to continue warfarin or switch to a DOAC. Participants who switched to DOACs were 69% more likely to experience either a major or clinically relevant nonmajor bleeding complication within 12 months, with similarly low rates of thromboembolism in both arms.

Many older adults with frailty may be eligible for low-dose anticoagulation, as the FDA indicates dose reduction for individuals with two or more of the following characteristics: age ≥ 80 years, body weight ≤ 60 kg, or serum creatinine ≥ 1.5 mg/dL [94]. The landmark study supporting low-dose anticoagulation—the randomized, placebo-controlled ELDERCARE-AF trial—included Japanese older adults with nonvalvular AF and age ≥ 80 years who were not considered appropriate candidates for full-dose oral anticoagulation, either due to critical bleeding, creatinine clearance of 15–30 mL per minute, or continuous use of NSAID or antiplatelet drugs [37]. Of these individuals, 40.9% of whom were frail, those who were randomized to receive low-dose edoxaban (15 mg daily) had reduced rates of stroke or systemic embolism compared with placebo without significantly increased incidence of major bleeding. A secondary analysis demonstrated that, regardless of frailty status, low-dose edoxaban reduced rates of stroke and systemic embolism without significantly increasing bleeding risk and, moreover, found no interaction between frailty and the association of edoxaban and bleeding [95].

Alternately, the 2023 US AF guideline considers percutaneous left atrial appendage to be reasonable (Class 2A) for individuals with moderate to high risk of stroke and a non-reversible contraindication to oral anticoagulation, including serious bleeding due to recurrent falls related to a non-reversible cause [1]. Occlusion of the left atrial appendage, the anatomic location most prone to thrombus formation due to AF-related stasis, has demonstrated similar rates of thromboembolism prevention compared with warfarin, with reduced risk of major bleeding [96]. This may represent an appealing strategy for frail older adults at high risk of fall-related bleeding; however, recent studies have demonstrated that, among older adults undergoing percutaneous left atrial appendage occlusion, frailty is associated with more procedural complications and higher 30-day and 1-year mortality rates [97,98,99]. Moreover, although older adults generally derive similar benefits from percutaneous left atrial appendage occlusion compared with younger adults, life expectancy should be taken into account in shared decision-making, as the long-term bleeding risk reduction may not outweigh the short-term procedural risk until approximately 2 years post-intervention [100,101]. Notably, surgical left atrial appendage occlusion is indicated (Class 1A) for patients with AF and moderate-to-high stroke risk who are undergoing cardiac surgery for another reason [1]. Thus, AF screening is reasonable for any patient with frailty undergoing coronary artery bypass graft surgery, and for surgical candidates with known AF, the medical team should ensure the cardiac surgery team is aware of the patient’s comorbid AF.

In the coming years, factor XI and XIa inhibitors, such as abelacimab and melvexian, represent an exciting therapeutic strategy for thromboembolism prevention in older adults at high risk of bleeding. Mechanistically, DOACs result in decreased activity of factor X, with warfarin—a vitamin K antagonist–additionally impairing factors II, VII, IX, and X. As these factors are all essential in thrombus formation, their inhibition reduces the risk of pathologic thrombus but also impairs appropriate hemostatic thrombus formation, thereby increasing the risk of bleeding. Unlike the aforementioned coagulation factors, factor XI participates in the growth of thrombi but not their formation [102]. Pre-clinical studies have shown, as anticipated, that factor XI inhibition impairs pathologic thrombus formation without increasing bleeding risk [103,104]. The results of two phase 2 trials of factor XI/XIa inhibitors have thus far been released, with both finding significantly lower bleeding risk compared with a DOAC, with near-complete inhibition of factor XI activity [105,106]. Although factor XI inhibition represents a promising alternative to low-dose DOAC and left atrial appendage occlusion for older adults with frailty and high bleeding risk, costs may be prohibitive for years after approval unless policies are developed to ensure broad access to this class of medications. Table 4 shows some of the main studies referenced throughout this manuscript. 

## 8. Conclusions

As the population ages and the prevalence of AF and frailty increases, improving evidence-based assessment and management of patients with both conditions is a growing clinical, investigational, and public health imperative [5]. The 2023 US AF guideline, while paradigm-shifting, does not explicitly address patients with frailty, marks a vital opportunity to reassess the current state of evidence for this high-risk patient population [1].

Given the physiologic connections and shared risk factors between AF and frailty, systematic AF screening may be considered for patients with frailty, and frailty testing may be incorporated into the evaluation of older adults with atrial fibrillation [1,2,9,32,35]. The staples of AF medical management—rate control, early rhythm control, and oral anticoagulation—are challenging for patients with frailty due to polypharmacy, multimorbidity, and concern for adverse effects, resulting in under-prescription of indicated medications [2,89,107]. Alternatives such as catheter ablation, percutaneous left atrial appendage occlusion, and low-dose anticoagulation all have limited evidence for frail older adults and merit further study [80,97,105].

To reduce the morbidity and mortality associated with comorbid AF and frailty in the coming years, clinicians and researchers can incorporate routine frailty assessment and management for patients with AF, and public health leaders can work to improve access to necessary therapies and resources for this complex patient population [32,108].

## Figures and Tables

**Table 1:** Stages of AF According to the 2023 Joint Atrial Fibrillation Guideline [1].

AF Stage	Name	Definition
1	At risk for AF	Presence of AF risk factors
2	Pre-AF	Structural or electrical findings predisposing to AF
3A	Paroxysmal AF	Intermittent AF, lasting up to 7 days
3B	Persistent AF	Continuous and sustained AF for more than 7 days requiring intervention
3C	Long-standing persistent AF	Continuous AF lasting > 12 months
3D	Successful AF ablation	Free from AF after ablation or surgical intervention
4	Permanent AF	No further attempts at rhythm control

**Table 2:** Rate Control Agents in Older Adults with Frailty.

Medication Class	Important Adverse Effects	Considerations in Presence of Frailty
Beta Blockers	Hypotension Negative cardiac inotropy Fatigue Increased airway resistance/bronchospasm Confusion Sleep disturbance	Potential interaction with topical beta blockers used for glaucoma Potential interaction with acetylcholinesterase inhibitors Atenolol is cleared by renal elimination
Calcium Channel Blockers	Hypotension Negative cardiac inotropy Peripheral edema Constipation Fatigue Dyspnea Flushing Tachycardia	Contraindicated in the presence of systolic HF Edema can lead to exacerbation of baseline inactivity
Digoxin	Cardiac arrhythmia (accelerated junctional rhythm) Visual disturbance	Increased risk for toxicity in older adults, those with renal impairment, amyloidosis, and low body weight Rarely used as monotherapy

**Table 3:** Antiarrhythmic Medications in Older Adults with Frailty.

Antiarrhythmic	Class	Elimination	Adverse Effects	Frailty Considerations				
				Use in Structural Heart Disease	Dosage Adjustments for Renal Function	Screening for Fall Risk	Screening for Drug Interactions	Drug Monitoring
Amiodarone	III	Liver	AV block Bradycardia Prolonged QT interval Torsades de pointes Corneal deposits Hepatotoxicity Hyper/hypothyroidism Pulmonary toxicity Nausea/Vomiting Photosensitivity	✔	×	✔	✔	TSH LFTs EKG CXR and PFTs
Dofetilide	III	Kidney	Bradycardia Prolonged QT interval Torsades de pointes	✔	✔	✔	✔	EKG (and telemetry for 3 days during initiation) Electrolytes Creatinine
Flecainide	I	Liver (70%) Kidney (30%)	QT prolongation AV Block Atrial flutter Ventricular tachycardia HFrEF exacerbation Dizziness Nausea Visual disturbances	×	✔	✔	✔	EKG
Propafenone	I	Liver	Bradycardia AV Block Atrial flutter Ventricular tachycardia HRrEF exacerbation Dizziness Nausea and taste disturbances Visual disturbances	×	×	✔	✔	EKG
Sotalol	III	Kidney	Bradycardia AV Block Prolonged QT interval Torsades de pointes HFrEF exacerbation Bronchospasm GI upset	✔	✔	✔	✔	EKG Electrolytes Creatinine
Dronedarone	III	Liver	Bradycardia Prolonged QT interval Torsades de pointes GI upset Fatigue/weakness	×	×	✔	✔	EKG LFTs
Catheter Ablation	N/A	N/A	Bleeding complications Infection risk General anesthesia risks Thromboembolic event Cardiac perforation Post-ablation syndrome	✔	×	×	×	EKG

Abbreviations: AV block, atrioventricular block; TSH, Thyroid stimulating hormone; LFTs, Liver function tests; EKG, electrocardiogram; HFrEF, Heart failure with reduced ejection fraction; PFTs, pulmonary function tests.

**Table 4:** Evidence-based studies that support the assessment and management of older adults with AF.

Study	Setting	Study Design	Intervention	Primary Outcome	Results
Hobbs et al. [44]	UK	Randomized controlled trial	AF screening (opportunistic and systematic) in adults aged ≥ 65	Incidence of new cases of AF and incremental cost per case detected	AF screening increased new AF detection rates
Svendsen et al. [49]	Denmark	Randomized controlled trial	AF screening in adults aged 70–90 with at least one stroke risk factor	Time to first stroke or systemic arterial embolism	Loop recorder increased AF detection
Wyse et al. [57]	US and Canada	Randomized controlled trial	Rate control vs. rhythm control in adults aged ≥ 65 with AF	Overall mortality	No survival advantage between rhythm and control and rate control
Van Gelder et al. [59]	Netherlands	Randomized controlled non-inferiority trial	Lenient rate control vs. strict rate control in adults age ≤ 80 with permanent AF	Composite of death from cardiovascular causes, hospitalization for heart failure, stroke, systemic embolism, bleeding, and life-threatening arrhythmic events	Lenient rate control was non-inferior to the prevention of the primary outcome
Kirchhof et al. [75]	European countries	Randomized, open-label trial with blinded-outcome trial	Early rhythm control vs. usual care in asymptomatic and symptomatic adults with AF	Composite of death from cardiovascular causes, stroke, or hospitalization with worsening of heart failure or acute coronary syndrome	The rhythm-control strategy had a lower risk of the primary outcome
Packer et al. [79]	10 Countries	Randomized controlled trial	Catheter ablation vs. drug therapy in adults with AF	Composite of death, disabling stroke, serious bleeding, or cardiac arrest	No difference in the primary outcome
Kim et al. [25]	Korea	Retrospective cohort study	N/A	First occurrence of ischemic stroke, major bleeding, or cardiovascular death	Oral anticoagulants in frail adults with AF decreased the risk of the primary outcome
Okumura et al. [37]	Japan	Randomized, double-blind, placebo-controlled trial	Low dose Edoxaban vs. placebo in adults age ≥ 80 with AF	Primary efficacy endpoint: composite of stroke or systemic embolism. Primary safety endpoint: major bleeding	Low-dose Edoxaban decreased the risk of stroke or systemic embolism with no increased risk of major bleeding
